# Complete Genome Sequence of “*Candidatus* Thioglobus sp.” Strain NP1, an Open-Ocean Isolate from the SUP05 Clade of Marine *Gammaproteobacteria*

**DOI:** 10.1128/MRA.00097-19

**Published:** 2019-03-14

**Authors:** Rachel L. Spietz, Katharine T. Marshall, Xiaowei Zhao, Robert M. Morris

**Affiliations:** a School of Oceanography, University of Washington, Seattle, Washington, USA; b University of Texas Southwestern Medical Center, Dallas, Texas, USA; University of Southern California

## Abstract

“*Candidatus* Thioglobus sp.” strain NP1 is an open-ocean isolate from the SUP05 clade of *Gammaproteobacteria*. Whole-genome comparisons of strain NP1 to other sequenced isolates from the SUP05 clade indicate that it represents a new species of SUP05 that lacks the ability to fix inorganic carbon using the Calvin-Benson-Bassham cycle.

## ANNOUNCEMENT

Members of the SUP05 clade of *Gammaproteobacteria* have an important role in carbon, nitrogen, and sulfur cycling (1, 2). The clade comprises diverse members, including chemoautotrophs that are abundant in suboxic and anoxic waters enriched in sulfide (1, 3, 4) and mixotrophic members that are ubiquitous in seawater (5). We sequenced the genome of “*Candidatus* Thioglobus sp.” strain NP1, a member of the SUP05 clade that was isolated from the deep chlorophyll maximum (45 m) in the North Pacific subpolar gyre by high-throughput dilution to extinction on natural seawater media, as previously described (6). Cells for sequencing were inoculated from a single starter culture and grown in 20 200-ml cultures in 250-ml polycarbonate flasks containing filter-sterilized seawater amended with 1 mM thiosulfate and a cellular lysate derived from the marine diatom Thalassiosira pseudonana CCMP 1335. All cultures were grown at the *in situ* temperature of 13°C in the dark under aerobic growth conditions. Cells were filtered through sterile 0.2-μm Supor 200 polyethersulfone filters when cultures reached early stationary phase (∼1 × 10^6^ cells/ml). DNA was then extracted and purity was verified by terminal restriction fragment length polymorphism analysis following previously described methods (6). Remaining DNA was purified on a single PowerClean DNA cleanup column (Mo Bio Laboratories, Carlsbad, CA, USA). A total of 2.25 µg of high-quality purified DNA was recovered and submitted to the University of Washington’s Genome Science Department’s Pacific Biosciences Services lab (http://pacbio.gs.washington.edu). A PacBio single-molecule real-time (SMRT) 10-kb library was constructed and sequenced using a single SMRT cell on the PacBio RS II platform. A total of 351,655 subreads were acquired, with an *N*_50_ value of 3,817 bp. Subreads shorter than 50 bp or with a quality score below 75 were removed prior to assembly. *De novo* assembly of the genome from reads was conducted using Hierarchical Genome Assembly Process 2 (HGAP2) (7). Through the HGAP2 pipeline, reads were preassembled using BLASR (8), assembled using Celera (9), and polished using the Quiver consensus algorithm (7) with default settings. The SMRT Analysis pipeline produced a single linear contig with overlapping ends and mean coverage of 564×. Genome ends were aligned and trimmed in Geneious v7.1.9 to produce a single 1.69-Mbp circular chromosome. The complete, finished genome of strain NP1 was annotated using NCBI’s Prokaryotic Annotation Pipeline.

Strain NP1 has a single circular genome 1,689,404 bp long, codes for 1,738 genes, and has a GC content of 35%. The average nucleotide identities using BLAST+ (ANIb) between “*Ca*. Thioglobus sp.” strain NP1 and other cultured SUP05 clade strains (PS1 and EF1) were calculated using JSpeciesWS (10). The ANIb between strain NP1 and strain PS1 was 75%. The ANIb between strains NP1 and EF1 was 67%. These ANIb values are well below the accepted 94 to 95% similarity cutoff values typically used for species classification (11, 12). The *in silico* DNA-DNA hybridization (DDH) values between strain NP1 and strains PS1 and EF1 were estimated using formula 2 in the Genome-to-Genome Distance calculator v2.1 (13). The *in silico* DDH values between strain NP1 and strains PS1 and EF1 were 18.9% and 18.6%, respectively. This is well below 70%, the value typically used for species classification (14).

### Data availability.

The complete genome sequence of “*Candidatus* Thioglobus sp.” strain NP1 is available in GenBank under accession number CP023860. Run data are available from the Sequence Read Archive (SRA) under the accession number SRR8501634.
